# Depletion of PHD3 Protects Heart from Ischemia/Reperfusion Injury by Inhibiting Cardiomyocyte Apoptosis

**DOI:** 10.1016/j.yjmcc.2015.01.007

**Published:** 2015-01-26

**Authors:** Liang Xie, Xinchun Pi, Zhongjing Wang, Jun He, Monte S. Willis, Cam Patterson

**Affiliations:** 1 Cardiovascular Research Institute, Department of Medicine Baylor College of Medicine, Houston, TX 77030; 2 UNC McAllister Heart Institute University of North Carolina, Chapel Hill, NC 27599; 3 Department of Pathology & Laboratory Medicine University of North Carolina, Chapel Hill, NC 27599; 4 NewYork-Presbyterian Hospital/Weill-Cornell Medical Center, New York, NY 10065

**Keywords:** Prolyl-4 hydroxylase domain protein, hypoxia inducible factor, ischemia-reperfusion, oxidative stress, DNA damage response

## Abstract

PHD3, a member of a family of Prolyl-4 Hydroxylase Domain (PHD) proteins, has long been considered a pro-apoptotic protein. Although the pro-apoptotic effect of PHD3 requires its prolyl hydroxylase activity, it may be independent of HIF-1α, the common substrate of PHDs. PHD3 is highly expressed in the heart, however, its role in cardiomyocyte apoptosis remains unclear. This study was undertaken to determine whether inhibition or depletion of PHD3 inhibits cardiomyocyte apoptosis and attenuates myocardial injury induced by ischemia-reperfusion (I/R). PHD3 knockout mice and littermate controls were subjected to left anterior descending (LAD) coronary artery ligation for 40 minutes followed by reperfusion. Histochemical analysis using Evan’s Blue, triphenyl-tetrazolium chloride and TUNEL staining, demonstrated that myocardial injury and cardiomyocyte apoptosis induced I/R injury were significantly attenuated in PHD3 knockout mice. PHD3 knockout mice exhibited no changes in HIF-1α protein level, the expression of some HIF target genes or the myocardium capillary density at physiological condition. However, depletion of PHD3 further enhanced the induction of HIF-1α protein at hypoxic condition and increased expression of HIF-1α inhibited cardiomyocyte apoptosis induced by hypoxia. In addition, it has been demonstrated that PHD3 plays an important role in ATR/Chk1/p53 pathway. Consistently, a prolyl hydroxylase inhibitor or depletion of PHD3 significantly inhibits the activation of Chk1 and p53 in cardiomyocytes and the subsequent apoptosis induced by doxorubicin, hydrogen peroxide or hypoxia/re-oxygenation. Taken together, these data suggest that depletion of PHD3 leads to increased stabilization of HIF-1α and inhibition of DNA damage response, both of which may contribute to the cardioprotective effect seen with depletion of PHD3.

## 1. Introduction

Despite improvements in diagnosis and medical treatment, ischemic heart disease remains the leading cause of morbidity and mortality in the United States [1]. Heart failure resulting from myocardial infarction (MI) and reperfusion accounts for over two-thirds of all cases of heart failure in the United States [2]. Strategies designed to preserve functional myocardium after MI, such as reducing myocardial cell death or stimulating angiogenesis [3, 4], are therefore crucial for reducing the incidence of heart failure. Although hundreds of experimental interventions have been demonstrated to be cardioprotective in preclinical studies, most of them fail to be translated into clinical practice [5]. Nonetheless, the recent focus on activating the hypoxia-inducible factor (HIF) pathway, either by inhibiting HIF-1α degradation or by overexpressing HIF-1α, in the heart may show promise as a possible therapeutic avenue for treating ischemic heart disease [6, 7].

HIF-1 is the principle transcription factor involved in the adaptive response to hypoxia. HIF is a heterodimer comprised of an alpha subunit, which is very unstable under normoxic condition, and a beta subunit. During hypoxia, HIF-1α accumulates and forms a heterodimer with HIF-β to activate over 200 genes, many of which are involved in the regulation of cell survival, anaerobic metabolism and angiogenesis [8]. However, under normoxic condition, HIF-1α is hydroxylated resulting in its ubiquitination and subsequent proteasomal degradation [9, 10]. A family of Fe^+2^ and 2-oxoglutarate-dependent dioxygenases, termed Prolyl-4 Hydroxylase Domain (PHD) 1–3 proteins, is responsible for the hydroxylation of HIF-1α [10, 11]. Interestingly, both PHD2 and 3 are highly expressed in the heart [12]. Cardiac-specific knockdown or knockout of PHD2, the major HIF-1α prolyl-4 hydroxylase in the heart, increases the myocardium capillary density and protects mice from myocardial injury induced by ischemia or I/R [13–15]. PHD3 has been suggested to play a compensative role in the regulation of HIF-1α stability, especially when PHD2 is absent or under ischemic conditions [16, 17]. Knocking down the expression of PHD3 significantly increases the capillary density in ischemic hindlimb and improves blood perfusion to the ischemic foot [17]. In addition to its regulatory role in HIF pathway, PHD3 has long been considered a pro-apoptotic protein and regulates apoptosis of a wide variety of cell types in HIF-independent pathways [12, 18, 19]. Notably, we have recently demonstrated that PHD3 plays an important role in the DNA Damage Response (DDR) and apoptosis induced by DNA damage [20]. However, its role in cardiac function remains unclear. Based on these recent findings, we hypothesize that PHD3 may play a crucial role in cardiomyocyte apoptosis and depletion or inhibition of PHD3 may also be cardioprotective.

## 2. Materials and methods

### 2.1. Cell culture

HL-1 cardiomyocytes (derived from murine atrial cardiomyocytes) were maintained according to previously published methods [21]. Primary neonatal rat or mouse ventricular cardiomyocytes were isolated from 1-2-day-old rats or mice using neonatal cardiomyocyte isolation kit (Worthington-biochem) and maintained in MEM medium supplemented with 10% horse serum, 5% fetal bovine serum, antibiotics (100 U/ml penicillin, 68.6 mol/L streptomycin) and BrdU (100 μM). With this protocol, over 95% of cells remained are cardiomyocytes as determined by staining with MF-20 antibody that reacts with sarcomere myosins. pO_2_ was controlled by incubating cells at 37°C in humidified, O_2_/CO_2_-regulated incubator (Coy Laboratory Products) adjusted to 5% CO_2_ and the indicated pO_2_.

### 2.2. Western-blot, immunofluorescence and TUNEL staining

After appropriate treatments, cells were washed with PBS and harvested in lysis buffer (1% Triton X-100, 50 mmol/L Tris, pH 7.4, 150 mmol/L NaCl, protease and phosphatase inhibitors). Cell lysates were clarified by centrifugation at 16,000g for 10 minutes. Equal amounts of protein were immunoblotted with corresponding antibodies as described.

Freshly isolated hearts were fixed with 10% formalin and embedded in paraffin. Heart sections were mounted on glass slides and then de-paraffinized/hydrated for immunostaining or TUNEL staining. Cultured cells fixed in 4% paraformaldehyde or tissue slides were permeabilized with 0.2% Triton X-100 for 5 minutes at room temperature. After washing in PBS, the cells or slides were sequentially treated with 5% goat serum for 1 hour (for blocking), then with the primary antibodies overnight in the blocking solution. After 3 washes, cells or slides were incubated in the dark with a secondary antibody conjugated to Alexa Fluor 488 (Molecular Probes, Eugene, OR) in blocking solution for 60 minutes at room temperature and then counterstained with DAPI. TUNEL staining was performed following the manufacturer’s protocol (S7100, Millipore, MA). Images were taken with fluorescence microscopy or confocal laser scanning microscopy.

### 2.3. Caspase 3/7 activity assay

Caspase 3/7 activity was measured using a Caspase-Glo® 3/7 Assay according to the manufacturer’s instructions (Promega; Madison, WI). In brief, HL-1 cells were cultured in a 24-well plate. Following transfection of Si-Chk1 or scramble Si-RNA control for 2 days, cells were then treated with 1mM doxorubicin for 8 hours. After treatment, 100 μL of Caspase-Glo® 3/7 Reagent was added to each well for luminescence measurement.

### 2.4. Real-time PCR

cDNA was synthesized from 1 μg RNA purified from heart tissues using iScript^™^ cDNA synthesis kit (Bio-Rad, Hercules, CA, US). Gene-specific mRNA levels were measured using the LightCycler® 480 Real-Time PCR system (Roche Diagnostic Co.-Roche Applied Science, Indianapolis) with light cycler 480 probe master (Roche Diagnostic Co.) and their specific primers and probes (designed by Roche Universal ProbeLibrary Assay Design Center software at https://www.roche-applied-science.com/sis/rtpcr/upl/index.jsp?id=UP030000). Samples were analyzed in triplicate with each PCR reaction containing 2μl cDNA (diluted 10-fold), 6.25 μl master mix and 0.375 μl primer-probe. Results are expressed as the ratio of gene of interest corrected to the housekeeping gene mRNA levels and then normalized to wildtype. The primer sequences were: phosphofruktokinase 1 (Pfkl) forward, ATGGATCCCAGCAGCAAG and reverse, CCAGTGTTATAGCCGAACTGC; pyruvate dehydrogenase kinase 1 (PDK-1) forward, GTTGAAACGTCCCGTGCT and reverse, GCGTGATATGGGCAATCC; Phosphoglycerate kinase (PGK) forward, TACCTGCTGGCTGGATGG and reverse, CACAGCCTCGGCATATTTCT; BCL2/adenovirus E1B 19-kDa protein-interacting protein 3 (Bnip3) forward, CCTGTCGCAGTTGGGTTC and reverse, GAAGTGCAGTTCTACCCAGGAG; PHD3 forward, CAGGTTATGTTCGCCATGTG and reverse, AGGACCCCTCCGTGTAACTT;

### 2.5 Si-RNA

Two sets of si-RNA oligos for mouse CHEK1 gene (Gene ID 12649) were ordered from Life Technologies. Catalog numbers are Chek1MSS202946 (UCCAGUAAAUAAUGGUUCCAGUGAA; UUCACUGGAACCAUUAUUUACUGGA) and Chek1MSS202948 (GAUUCUUUACUAAAUUGGAUGCGGA; UCCGCAUCCAAUUUAGUAAAGAAUC). Si-RNA oligos for mouse PHD3 gene (Gene ID, 112407) were purchased from Santa Cruz Biotechnoloy (sc-45800).

### 2.6 Hypoxia and hypoxia-reoxygenation treatment of cultured cells

For study of apoptosis induced by hypoxia, HL-1 cardiomyocytes were placed in a 0.5% O_2_ hypoxic glove box (Coy Laboratory) in serum-free media for 48 hours. For studies of signaling transduction induced by hypoxia-reoxygenation, HL-1 cardiomyocytes were placed in a 0.5% O_2_ hypoxic glove box in serum-free media for 6 hours. Cells were then removed from the hypoxic glove box and media were replaced with fresh serum-free media pre-equilibrated in a normoxic incubator. Cells were harvested at times indicated in the figures. For apoptosis induced by hypoxia-reoxygenation, neonatal mouse ventricular cardiomyocytes were transduced with adenoviruses expressing LacZ or Cre recombinase for three days and then placed in an “ischemic buffer” (118 mmol/L NaCl, 24 mmol/L NaHCO3, 1.0 mmol/L NaH2PO4, 2.5 mmol/L CaCl2-2H2O, 1.2 mmol/L MgCl2, 20 mmol/L sodium lactate, 16 mmol/L KCl, and 10 mmol/L 2-deoxyglucose, pH adjusted to 6.2) pre-equilibrated in a 0.5% O_2_ incubator [22]. After 1 hour, reoxygenation was obtained by replacing the ischemic buffer with normal serum-free media pre-equilibrated in a normoxia incubator overnight.

### 2.7 Animals

PHD3*^f^*^/^*^f^*; Cre^+/−^ and PHD3*^f^*^/^*^f^*; Cre^−/−^ mice were described previously [20]. Genotypes of these mice were determined by PCR described previously or using the protocol provided by Jackson laboratory [23]. PHD3*^f^*^/^*^f^*; Cre^+/−^ at 8~10 weeks old were intraperitoneally injected with tamoxifen (50 mg/kg/day) for five consecutive days to generate PHD3^−/−^ mice [24]. Depletion of PHD3 in hearts was confirmed by real-time PCR or Western-blot one week after the first injection of tamoxifen, using primers described previously [23]. Littermate PHD3*^f^*^/^*^f^*; Cre^−/−^ mice injected with tamoxifen were used as the controls. All animal protocols were reviewed and approved by the Institutional Animal Care Advisory Committee and were in compliance with the rules governing animal use as published by the National Institutes of Health.

### 2.8 LAD ischemia-reperfusion injury

Left anterior descending (LAD) coronary arteries of mice with indicated genotypes were tied for 40 minutes, followed by 1 or 3 days of reperfusion. Briefly, male mice (8–10 weeks old) were anesthetized with pentobarbital (45 mg/kg), intubated, and placed on a ventilator. The chest cavity was opened by an incision of the left fourth intercostal space, and the pericardial sac was removed to visualize the LAD coronary artery. A 7-0 silk suture was passed underneath the LAD artery ~1 to 2 mm below the left auricle and tied around a 1-mm length of polyethylene tubing (OD = 0.61 mm; Intramedic PE-10, Clay Adams, Parsippany, NJ) to produce myocardial blanching. After 40 minutes, blood flow was restored and the chest wall was then closed. Three days after reperfusion, the hearts were stained with Evan’s Blue to demarcate the area at risk (AAR) and with 2% triphenyl tetrazolium chloride (TTC) to identify infarcted area (IA). The area of IA and AAR were quantified using ImageJ (National Institutes of Health). For apoptosis analysis, 4 hearts from PHD3*^f^*^/^*^f^*; Cre^−/−^ and 6 hearts from PHD3*^f^*^/^*^f^*; Cre^+/−^ mice were fixed with 10% formalin and embedded with paraffin one day after reperfusion. Three sections from each heart were used for TUNEL staining. TUNEL positive cells from five views of each section in the area at risk were counted in a blinded manner.

### 2.9 Statistical Analysis

Data are presented as mean ± SEM. Differences between groups were evaluated for statistical significance using Student’s *t*-test. *P* values less than 0.05 were regarded as significant.

## 3. Results

### 3.1. Depletion of PHD3 attenuates ischemia-reperfusion injury and decreases cardiomyocyte apoptosis in vivo

**Figure 1:**
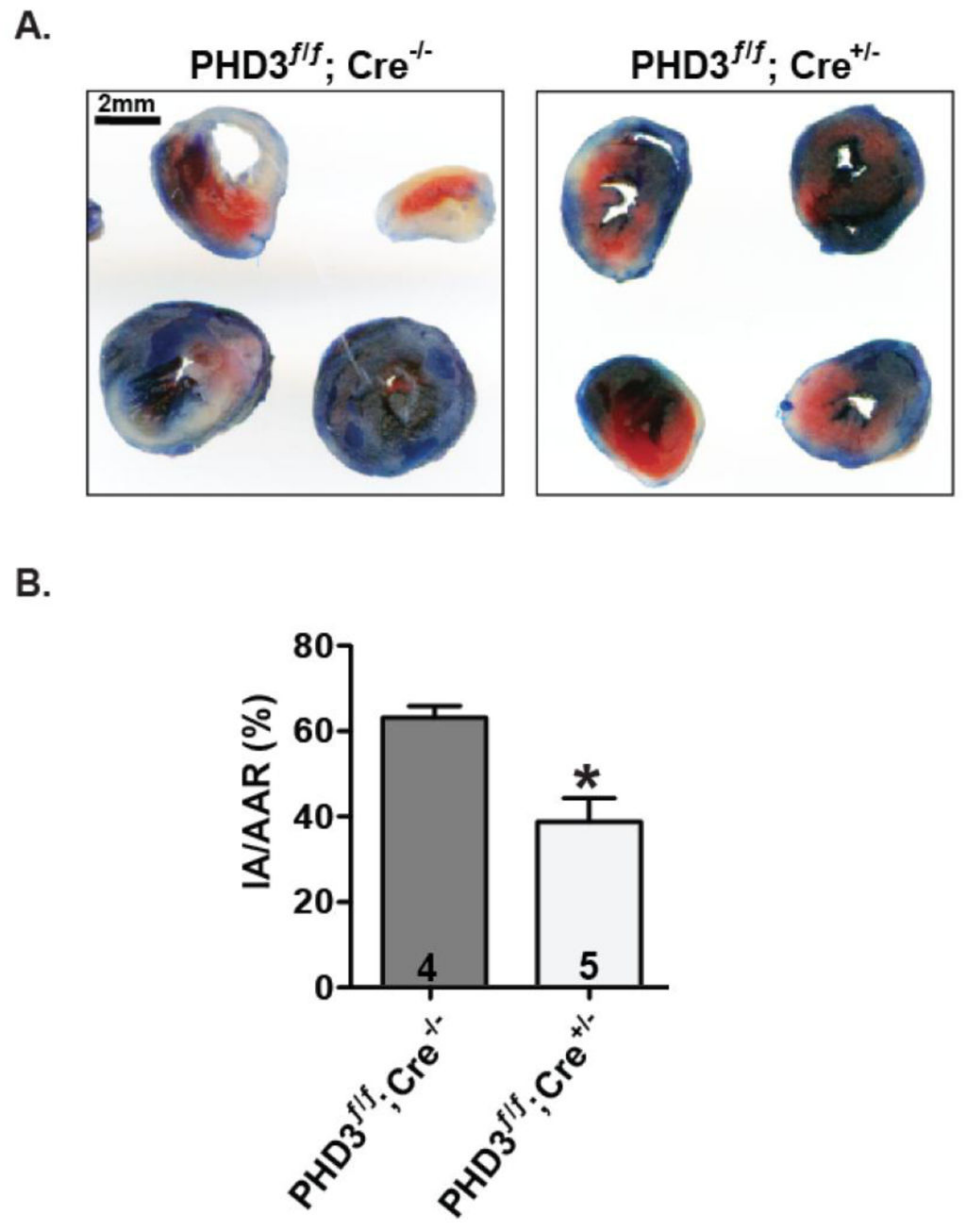
Depletion of PHD3 attenuates myocardial injury induced by I/R injury After 5 doses of tamoxifen, left anterior descending (LAD) coronary arteries of mice with indicated genotypes were tied for 40 minutes and then released for reperfusion. Three days after reperfusion, the hearts were stained with Evan’s Blue to demarcate the area at risk (AAR) and with 2% triphenyl tetrazolium chloride (TTC) to identify infarcted area (IA). Representative cross-sections of the stained hearts are shown in **(A)**. Quantitative analysis is shown in **(B)**. The numbers of mice analyzed are indicated in the bars respectively. *, p < 0.05.

**Figure 2:**
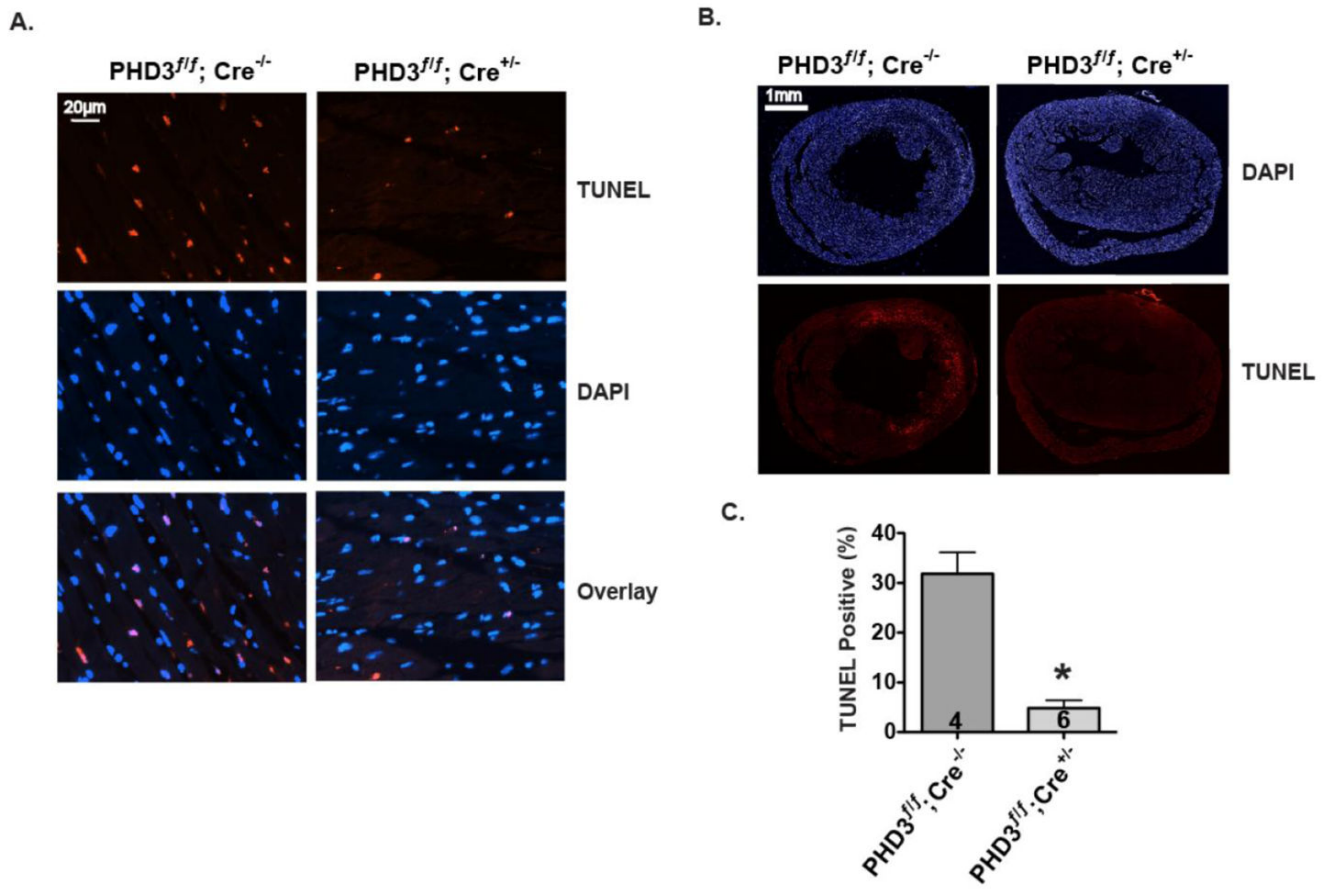
Depletion of PHD3 inhibits cardiomyocyte apoptosis induced by I/R injury After 5 doses of tamoxifen infusion, left anterior descending (LAD) coronary arteries of mice with indicated genotypes were tied for 40 minutes and then released for reperfusion. Twenty-four hours after reperfusion, hearts were fixed with 10% formaldehyde and embedded in paraffin. Cross sections of hearts were then analyzed with TUNEL staining. Nuclei were stained with DAPI. Representative high magnification images of the AAR are shown in **(A)** and low magnification images of whole sections are shown in **(B)**. Quantitative analysis of the apoptotic cells within the AAR is shown in **(C).** The numbers of mice analyzed are indicated in the bars respectively. *, p < 0.05. Data represent mean values ± SEM.

To examine whether depletion of PHD3 is cardioprotective, we generated PHD3*^f^*^/^*^f^*; Cre^+/−^ mice, which utilize a tamoxifen-inducible and Cre-mediated recombination system [20]. Depletion of PHD3 in the heart was obtained by intraperitoneal infusion of tamoxifen (50mg/kg/day) for five consecutive days. One week after the first injection of tamoxifen, myocardial injury was induced in PHD3*^f^*^/^*^f^*; Cre^+/−^ mice and the littermate controls (PHD3*^f^*^/^*^f^*; Cre^−/−^ mice) by subjecting them to ischemia for 40 minutes followed by reperfusion for 72 hours using a reversible ligation of the left anterior descending artery (LAD). Interestingly, although there were no gross abnormities observed in the hearts from PHD3*^f^*^/^*^f^*; Cre^+/−^ mice, the infarct area (IA) normalized by area at risk (AAR) in PHD3*^f^*^/^*^f^*; Cre^+/−^ mice was significantly smaller than that in the littermate controls (Figure 1A and B), suggesting that depletion of PHD3 protected hearts from ischemia-reperfusion (I/R) injury. In consistent with the pro-apoptotic role of PHD3 described in other cells [18–20], TUNEL staining revealed that cardiomyocyte apoptosis in the area of risk was also significantly decreased in PHD3*^f^*^/^*^f^*; Cre^+/−^ mice at 24 hours after reperfusion (Figure 2). Taken together, these results suggest that depletion of PHD3 may protect mice from ischemia-reperfusion injury by decreasing cardiomyocyte apoptosis.

### 3.2. Depletion of PHD3 further stabilizes HIF-1α at hypoxic condition, which may contribute to the cardioprotective effect

**Figure 3:**
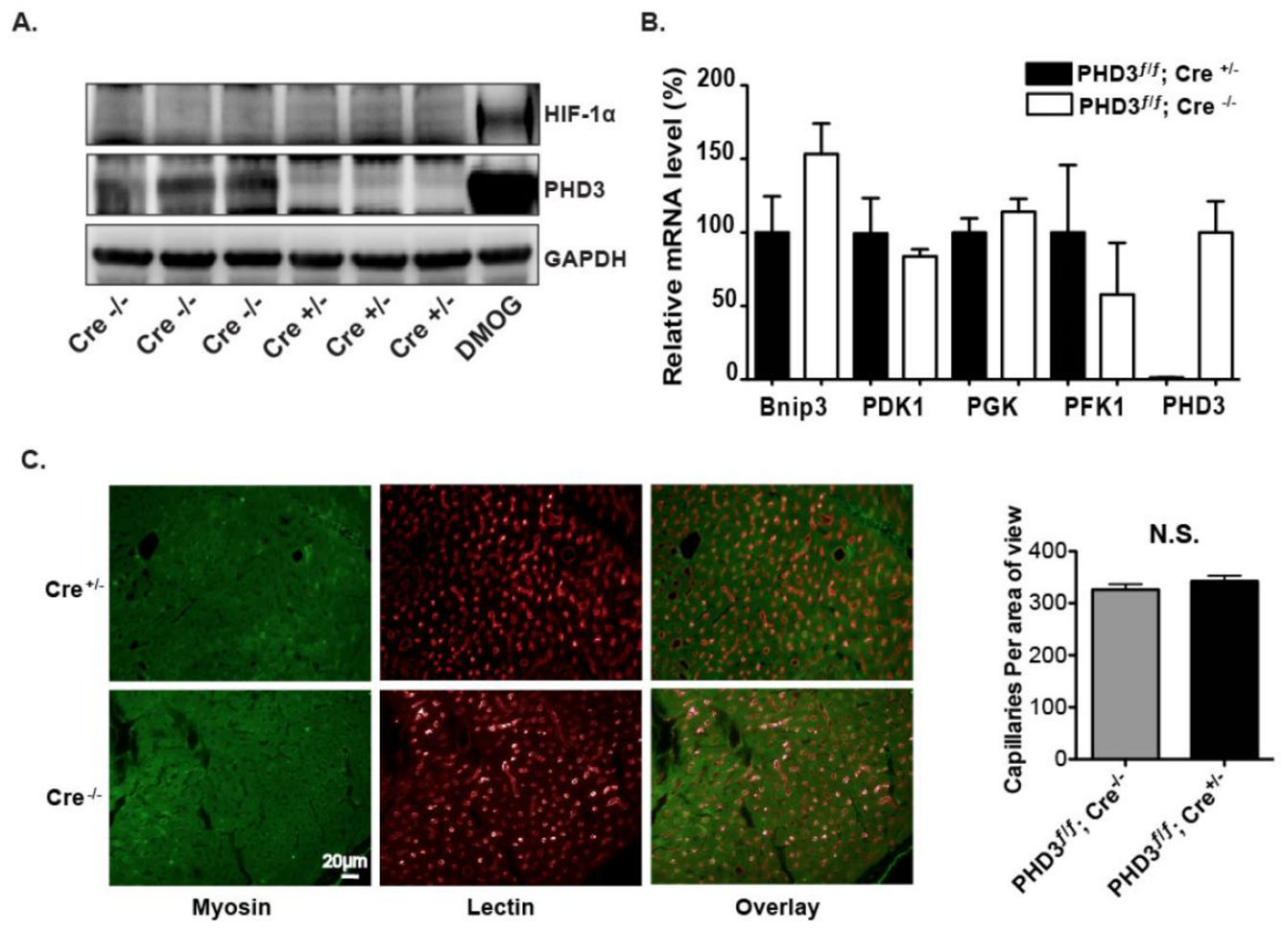
Depletion of PHD3 has no effect on HIF-1α protein level, the expression of HIF target genes or capillary density in the heart After 5 doses of tamoxifen, ventricles were excised and flash frozen. **(A)** Proteins extracted from ventricles of the indicated genotypes (n=3) were western-blotted with anti-HIF-1α and anti-PHD3 antibodies. Lysate from cardiomyocytes treated with DMOG (1mM) was used as the positive control for HIF-1α. **(B)** mRNAs were extracted from ventricles of the indicated genotypes. Relative mRNA level of HIF target genes and PHD3 were analyzed by quantitative real-time PCR. n = 3. **(C)** Heart sections of the indicated genotypes were immunostained with anti-myosin antibody and TRITC-E-lectin. Capillary densities are expressed as the number of lectin-positive objects per field of view. N.S., not significant, n = 3.

It is well documented that PHD2 is the major HIF-1α prolyl-4 hydroxylase in the heart and depletion of PHD3 alone has no effect on cardiac HIF-1α protein levels [15, 16]. Consistently, we didn’t observe any significant accumulation of HIF-1α protein in the hearts from PHD3*^f^*^/^*^f^*; Cre^+/−^ mice (Figure 3A). In addition, real-time PCR analysis demonstrated that there were no significant changes in the expression of some HIF target genes in the hearts of PHD3*^f^*^/^*^f^*; Cre^+/−^ mice either (Figure 3B), suggesting that HIF pathway is not further activated at physiological condition in PHD3*^f^*^/^*^f^*; Cre^+/−^ mice compared to PHD3*^f^*^/^*^f^*; Cre^−/−^ mice. As shown in figure 3A and B, we could barely detect both PHD3 protein and mRNA in the hearts of PHD3*^f^*^/^*^f^*; Cre^+/−^ mice, suggesting the successful deletion of PHD3 in the hearts. It was reported that knockdown of PHD2 increased the myocardium capillary density via HIF-1 pathway, which plays an important role in cardioprotection [13, 14]. Since we have not observed any significant activation of HIF-1 pathway in the heart of PHD3*^f^*^/^*^f^* null mice, we expect that depletion of PHD3 will not change the myocardium capillary density. To confirm this hypothesis, we analyzed the myocardium capillary density of PHD3*^f^*^/^*^f^*; Cre^+/−^ or PHD3*^f^*^/^*^f^*; Cre^−/−^ mice by immunostaining heart sections with anti-myosin and anti-E-lectin antibodies. As expected, we observed no significant difference in the myocardium capillary density between PHD3*^f^*^/^*^f^*; Cre^+/−^ and PHD3*^f^*^/^*^f^*; Cre^−/−^ mice (Figure 3C and D). Taken together, these results suggest that depletion of PHD3 has no effect on HIF-1 pathway and myocardium capillary density at physiological condition.

**Figure 4:**
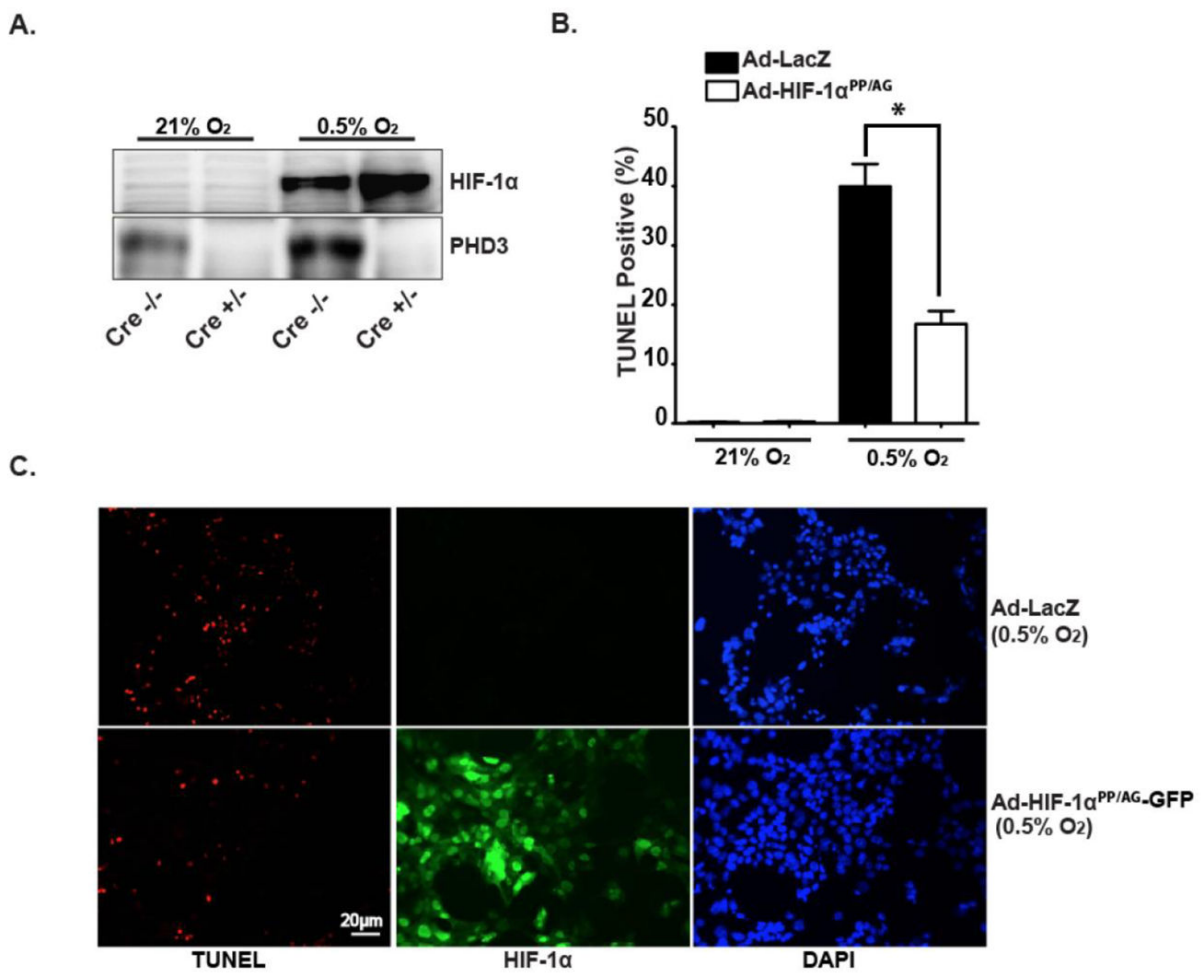
Depletion of PHD3 further stabilizes HIF-1α and overexpression of normoxia-stable HIF-1α protects cardiomyocytes from hypoxia-induced apoptosis **(A)** Neonatal ventricular myocytes from PHD3*^f^*^/^*^f^*; Cre^+/−^ or PHD3*^f^*^/^*^f^*; Cre^−/−^ mice were treated with 4-hydroxyl-tamoxifen for 3 days to delete PHD3. Cells were then cultured at 0.5% or 21% O_2_ condition for 8 hours. Cells were then harvested for western blots with the indicated antibodies. **(B), (C)** HL-1 cardiomyocytes were infected with adenovirus expressing normoxia-stable HIF-1α-GFP (HIF-1α^PP/AG^-GFP) or lacZ for 24 hours. Infected cells were then cultured with fresh serum-free medium at 0.5% or 21% O_2_ conditions for additional 48 hours to induce apoptosis. Cardiomyocytes were then fixed and stained with DAPI. Apoptosis was then analyzed with TUNEL staining. *, p < 0.01, n = 3. Representative images were shown in **(C)**.

However, it has been reported that PHD3 itself is a HIF-1 target and can be induced by hypoxia, which may serve as a form of negative feedback mechanism to regulate HIF-1α stability at hypoxic condition [10, 25]. Consistently, we demonstrated that activation of HIF-1 pathway by either prolyl hydroxylase inhibitor dimethyloxaloylglycine (DMOG) or hypoxia increased the expression of PHD3 (Figure 3A and 4A). More importantly, depletion of PHD3 in neonatal mouse ventricular cardiomyocytes further increased HIF-1α protein level at hypoxic condition (Figure 4A), suggesting that PHD3 can regulate HIF-1α stability at hypoxic condition. It is well documented that activation of the HIF-1 pathway can turn on a large number of genes, which are involved in the regulation of cell survival and metabolic reprogramming to fight against hypoxia-induced damage [8]. Increased expression of HIF-1α has also been shown to inhibit apoptosis in a variety of cell types [26, 27]. Therefore, we hypothesized that increased expression of HIF-1α may also protect cardiomyocyte from apoptosis induced by hypoxia. To examine this hypothesis, we overexpressed a normoxia-stable HIF-1α mutant, in which two proline residues at position 402 and 564 are replaced with Ala and Gly respectively [27], in HL-1 cells and then cultured them under hypoxic condition for 48 hours to induce apoptosis. Not surprisingly, we demonstrated that overexpression of HIF-1α^PP/AG^ significantly decreased the apoptosis of HL-1 cells cultured at hypoxic condition (Figure 4B and C). Taken together, we demonstrated that depletion of PHD3 further increased the protein level of HIF-1α at hypoxic condition and increased expression of HIF-1α protected cardiomyocytes from hypoxia-induced apoptosis.

### 3.3. Inhibition or depletion of PHD3 inhibits DNA damage response induced by doxorubicin in cardiomyocytes

It is generally accepted that oxidative stress plays a critical role in cardiomyocyte apoptosis induced by I/R injury [28]. Reactive oxygen species (ROS) induced by hypoxia-reoxygenation induces DNA damage and activates the DNA damage response (DDR) in human cancer cells and primary lymphocytes [29, 30]. However, whether oxidative stress induced by hypoxia/re-oxygenation also activates the DDR in cardiomyocytes, and how this might be mediated, is largely unclear. Interestingly, we have recently demonstrated that inhibition or depletion of PHD3 inhibits ATR/Chk1/p53 pathway and apoptosis induced by a wide variety of DNA damage agents, both in vitro and in vivo [20]. It is very interesting to examine whether PHD3 regulates the DDR and apoptosis induced by oxidative stress in cardiomyocytes.

**Figure 5:**
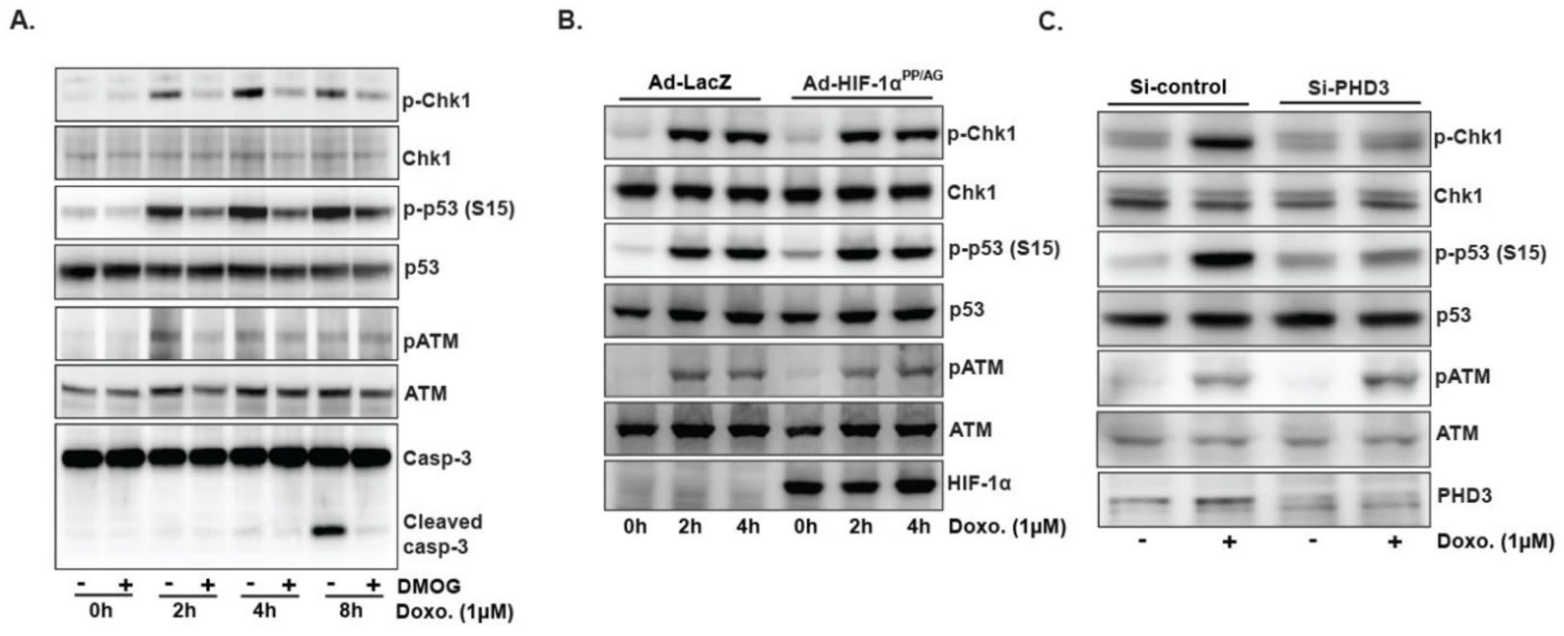
Inhibition or depletion of PHD3 inhibits DNA damage response induced by doxorubicin **(A)** HL-1 cells were pre-treated with DMOG for 4h and then treated with doxorubicin (1μM) as indicated. Western blots were performed with the indicated antibodies. **(B)** HL-1 cells were infected with adenovirus expressing LacZ or normoxia-stable HIF-1α^PP/AG^ for 24 hours. Cells were then treated with doxorubicin (1μM) and western blots were performed with the indicated antibodies. **(C)** After two days transfection with si-RNA as indicated, HL-1 cells were treated with doxorubicin (1μM) for 2 hours. Western blots were then performed with indicated antibodies.

Doxorubicin, a potent chemotherapy agent, causes cardiomyocyte apoptosis and cardiomyopathy[31]. It was demonstrated recently that oxidative DNA damage and subsequent activation of the DDR induced by doxorubicin played a pivotal role in this pathophysiological response [31, 32]. In order to better understand the role of PHD3 in the DDR and apoptosis induced by oxidative stress in cardiomyocytes, we first examined whether PHD3 is involved in the DDR induced by doxorubicin in cardiomyocytes. Consistent with previous reports, treatment of HL-1 cardiomyocytes with doxorubicin resulted in a robust activation of ATM/Chk1/p53 pathway and caspase-3 (Figure 5A). To examine the potential role of PHD3 in doxorubicin-induced DDR and apoptosis in cardiomyocytes, we pretreated HL-1 cells with dimethyloxaloylglycine (DMOG), a pan prolyl hydroxylase inhibitor, before treating them with doxorubicin. In consistent with our previous studies performed in other cell types [20], we demonstrated that DMOG dramatically inhibited the activation of both p53 and Chk1 and had no effect on the activation of ATM (Figure 5A and Figure S1A). Since depletion of PHD3 blocks the activation of Chk1 and p53 via ATR pathway but not ATM pathway[20], this result suggests that ATR, the other major upstream kinase of Chk1 and p53, is also activated by doxorubicin and PHD3 may also play a regulatory role in ATR/Chk1/p53 pathway in HL-1 cardiomyocytes. In addition, DMOG also dramatically inhibited the activation of caspase-3 induced by doxorubicin in HL-1 (Figure 5A).

Since HIF-1α can be stabilized by DMOG (Figure 3A), it is important to examine whether HIF-1α plays a role in the activation of Chk1/p53. Because HIF-1α protein is very unstable at normoxic condition, we overexpressed the normaxia-stable HIF-1α^PP/AG^ in HL-1 cells and then treated the cells with doxorubicin. As shown in figure 5B, overexpression of HIF-1α^PP/AG^ had no effect on the activation of Chk1 and p53, suggesting that the increased protein level of HIF-1α is not required for the inhibitory effect of DMOG on ATR/Chk1/p53 pathway. Finally, to confirm the specific role of PHD3 in ATR/Chk1/p53 pathway, we transfected HL-1 cells with si-PHD3 oligos to knock down the expression of endogenous PHD3 and demonstrated that PHD3 was crucial for the activation of Chk1 and p53 induced by doxorubicin in HL-1 cardiomyocytes (Figure 5C).

### 3.4. Inhibition of PHD3 decreases cardiomyocyte apoptosis induced by doxorubicin

**Figure 6:**
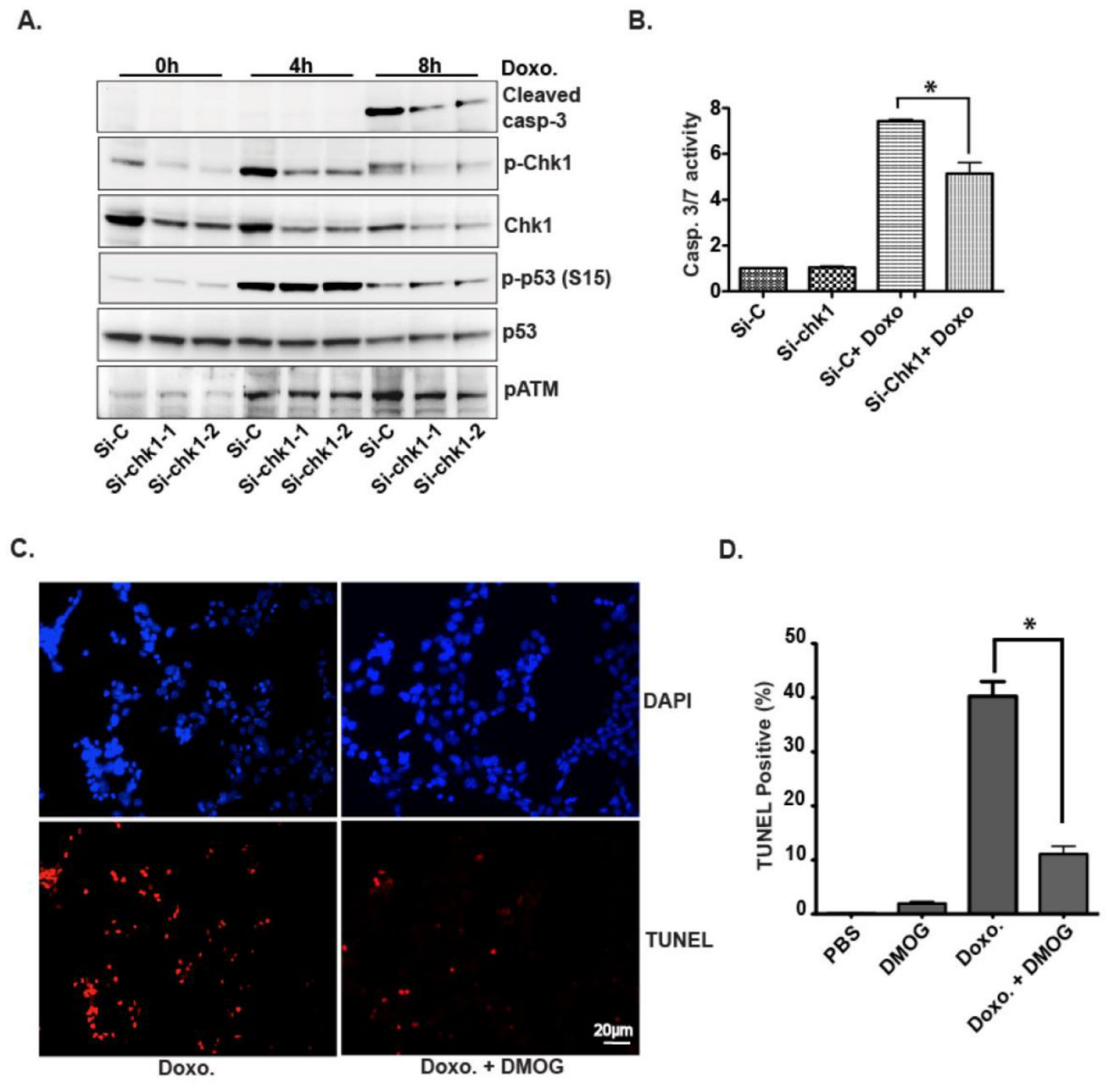
DMOG inhibits HL-1 cardiomyocyte apoptosis induced by doxorubicin **(A)** HL-1 cells were transfected with two sets of si-RNA for Chk1 or scramble si-RNA as the control (Si-C) for two days. Cells were then treated with doxorubicin (1μM) as indicated. Western blots were then performed with the indicated antibodies. **(B)** After two days transfection with si-RNAs, HL-1 cells were treated with doxorubicin (1μM) for 8 hours and then harvested for caspase3/7 activity assay. Knocking down the expression of Chk-1 significantly inhibits caspase3/7 activity. N = 3, *p < 0.05. **(C), (D)** HL-1 cells were treated with doxorubicin for 24 hours with or without pretreatment of DMOG. Apoptosis was then analyzed with TUNEL staining and nuclei were stained with DAPI. Quantitative analysis from 3 independent experiments is shown in **(D)**, *p < 0.05.

It was recently reported that DDR plays an essential role in cardiomyocyte apoptosis induced by doxorubicin [31]. Consistently, we demonstrated that knocking down the expression of Chk-1 dramatically inhibited the cleavage and activation of caspase-3 induced by doxorubicin in HL-1 cells, suggesting that the activation of Chk-1 is required for cardiomyocyte apoptosis induced by doxorubicin (Figure 6A and B). Furthermore, we performed TUNEL staining assay and demonstrated that DMOG, which can inhibit Chk1 activation (Figure 5A), significantly inhibited HL-1 cell apoptosis induced by doxorubicin (Figure 6C and D), suggesting that PHD3 enzymatic activity may be crucial for cardiomyocyte apoptosis induced by doxorubicin.

**Figure 7:**
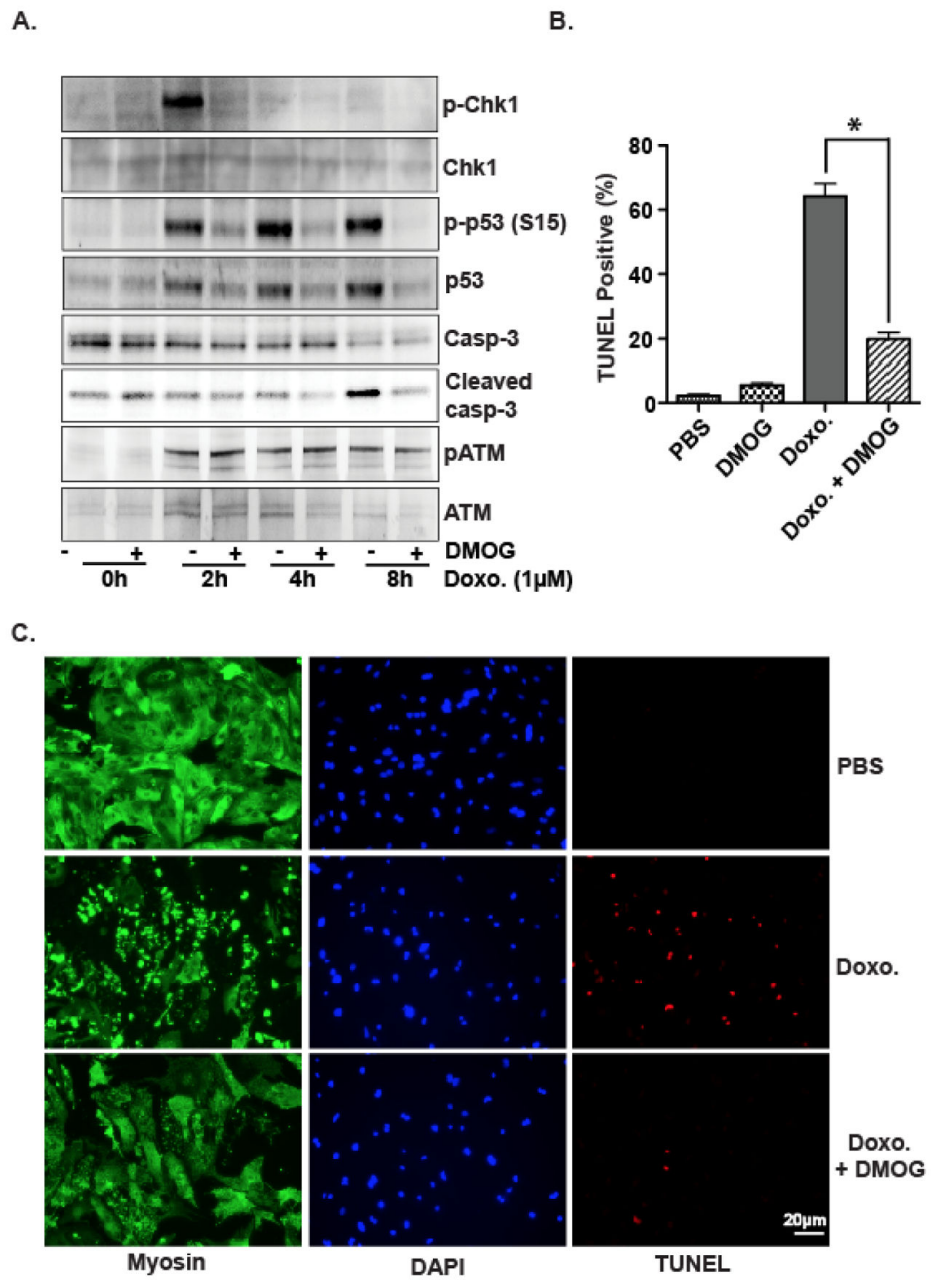
DMOG inhibits DNA damage response and apoptosis induced by doxorubicin in primary cardiomyocytes **(A)** Neonatal rat ventricular myocytes were pre-treated with DMOG for 4h and then treated with doxorubicin (1μM) as indicated. Western blots were performed with the indicated antibodies. **(B), (C)** Neonatal rat ventricular myocytes were treated with doxorubicin for 16h with or without pretreatment of DMOG. Cardiomyocyte apoptosis was then analyzed with TUNEL staining. Neonatal rat ventricular myocytes were also immunostained with MF20 antibody, which specifically recognizes myosin of striated muscle cells. Quantitative analysis is from 3 independent experiments. *p < 0.05. Representative images are shown in **(C)**.

To determine whether the inhibitory effects of DMOG on the DDR and apoptosis were also preserved in primary cardiomyocytes, we isolated neonatal rat ventricular myocytes (NRVM) and performed similar experiments described above. As shown in Figure 7A, the activation of p53, Chk1 and caspase-3 were markedly inhibited by DMOG in NRVM. In addition, doxorubicin-induced apoptosis of NRVM was also significantly inhibited in the presence of DMOG (Figure 7B and C). In summary, these data suggest that PHD3 may play a pivotal role in the DDR in cardiomyocytes. Inhibition or depletion of PHD3 inhibits Chk1/p53 activation and subsequent apoptosis caused by doxorubicin in cardiomyocytes.

### 3.5. PHD3 plays crucial role in the DDR and subsequent apoptosis induced by oxidative stress

**Figure 8:**
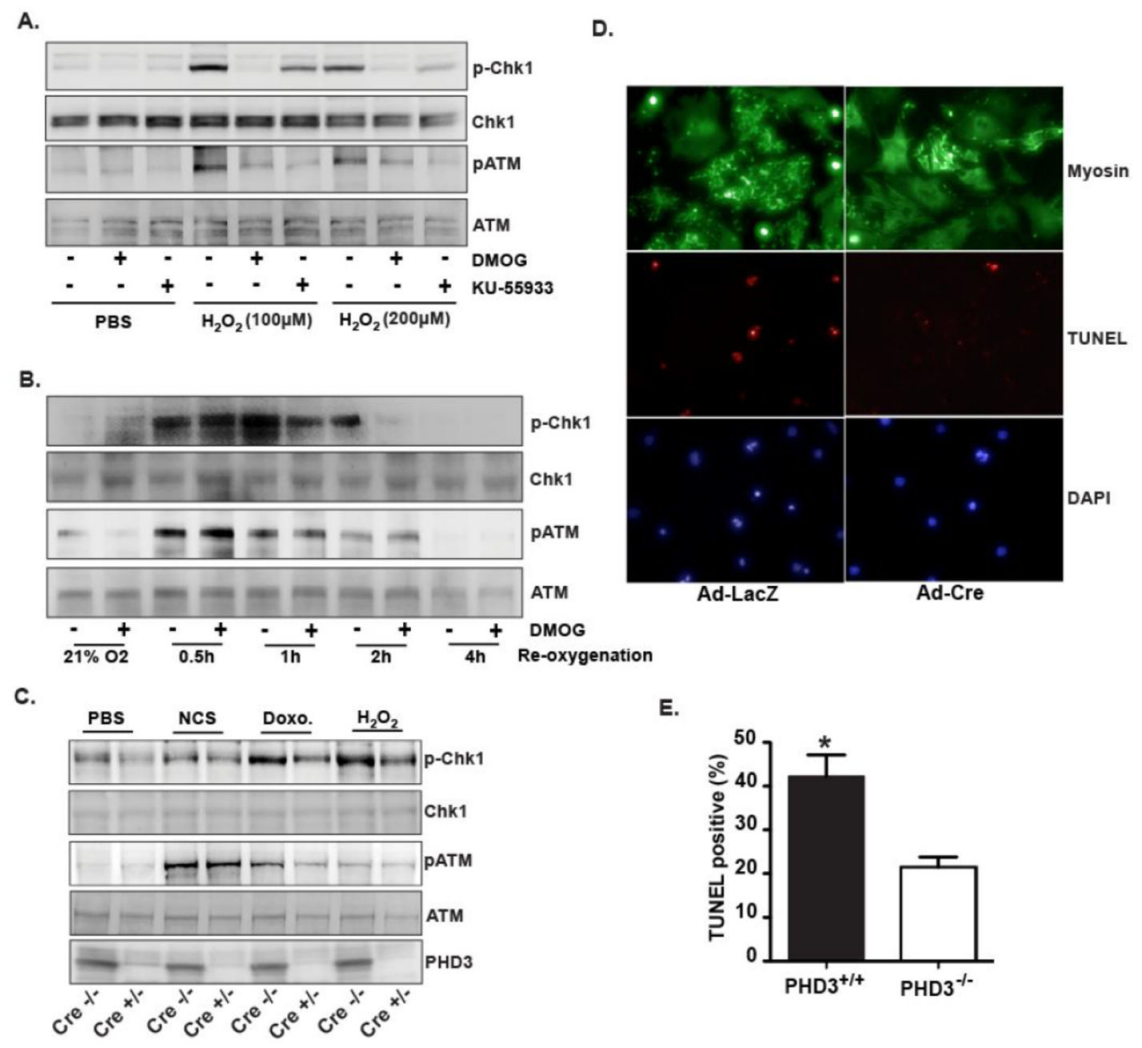
PHD3 plays a crucial role in DNA damage response and apoptosis induced by H_2_O_2_ or hypoxia-reoxygenation in cardiomyocytes **(A)** HL-1 cells were pre-treated with DMOG for 4h or KU55933 for 30 minutes and then treated with 100 μM or 200 μM H_2_O_2_ for 1 hour as indicated. Western blots were performed with the indicated antibodies. **(B)** HL-1 cells were cultured in a hypoxia chamber for 6 hours and then switched to normoxic conditions for the indicated time with or without pretreatment with DMOG. Western blots were performed with the indicated antibodies. **(C)** Neonatal mouse ventricular myocytes (NMVMs) from PHD3*^f^*^/^*^f^*; Cre^+/−^ or PHD3*^f^*^/^*^f^*; Cre^−/−^ mice were treated with 4-hydroxyl-tamoxifen for 3 days. Cells were then treated with NCS, Doxorubicin or H_2_O_2_ for 1h and western blots were performed with indicated antibodies. **(D), (E)** NMVMs from PHD3*^f^*^/^*^f^* mice were infected with adenovirus expressing cre recombinase or lacZ for 2 days. Infected cells were then cultured in ischemic medium at hypoxic condition for 1 hour. Re-oxygenation was obtained by culturing cells in normal medium at normoxic condition for 16 hours. Cells were immunostained with MF20 and apoptosis was analyzed with TUNEL staining. Quantitative analysis was shown in **(E)**. n=3, *p < 0.05.

To determine the direct role of ROS in the DDR in cardiomyocytes, HL-1 cells or NRVM were treated with H_2_O_2_ in the presence of DMOG or KU-55933, an ATM specific inhibitor. Not surprisingly, treatment of H_2_O_2_ led to a robust activation of ATM and Chk1 (Figure 8A). The activation of Chk1 was partially inhibited by KU55933, suggesting that ATM partially contributes to the activation of Chk1 induced by H_2_O_2_ in cardiomyocytes (Figure 8A). Interestingly, although DMOG had no effect on ATM activation induced by H_2_O_2_, it almost completely inhibited the activation of Chk1 in HL-1 cardiomyocytes (Figure 8A), suggesting ATR is the major upstream kinase for Chk1 activation induced by H_2_O_2_. It is well accepted that reperfusion injury is mainly caused by the oxidative stress invoked by re-oxygenation. To determine whether oxidative stress resulted from hypoxia/re-oxygenation activates the DDR in cardiomyocytes, we cultured HL-1 cells under hypoxic condition for 6 hours and then returned them back to normoxic condition for further culturing. As shown in Figure 8B and Figure S2, re-oxygenation strongly activated ATM/Chk1 whereas pretreatment of cells with DMOG dramatically inhibited the activation of Chk1. Taken together, these results suggest that the DDR can be activated by oxidative stress associated with H_2_O_2_ or hypoxia/re-oxygenation in cardiomyocytes and that PHD3 may play a pivotal role in this response.

To further define the role of PHD3 in the DDR and apoptosis in cardiomyocytes, neonatal mouse ventricular myocytes (NMVM) were isolated from PHD3*^f^*^/^*^f^*; Cre ^+/−^ and control littermates, and exposed to either doxorubicin or H_2_O_2_. As shown in Figure 8C and Figure S1B, depletion of PHD3 markedly inhibited Chk1 activation induced by doxorubicin or H_2_O_2_ but not ATM activation. Notably, although treatment of neocarzinostatin, which induces DNA double-strand break, strongly activated ATM, it barely activated Chk1 in NMVM. Finally, hypoxia/re-oxygenation induced a substantial amount of apoptosis of NMVM with wild type PHD3 (Figure 8D and E). As expected, depletion of PHD3 significantly inhibited the apoptosis induced by hypoxia/re-oxygenation (Figure 8D and E).

## Discussion

We have demonstrated for the first time that depletion of PHD3 protects the heart from myocardial injury and inhibits cardiomyocyte apoptosis induced by I/R in vivo or hypoxia in vitro in both HIF-1α-dependent and HIF-1α-independent pathways. Depletion or inhibition of PHD3 in cardiomyocytes further stabilizes HIF-1α at hypoxic condition and dramatically inhibits Chk1/p53 activation induced by doxorubicin, H_2_O_2_ or hypoxia/re-oxygenation, contributing to the cardioprotective effect seen with the depletion of PHD3.

Oxygen plays a key role in energy metabolism and is essential for the survival of aerobic organisms. Lack of oxygen will trigger a series of HIF-mediated adaptive responses [33]. Activation of the HIF pathway can turn on a large number of genes that are involved in the regulation of angiogenesis, vascular remodeling, cell survival and metabolic reprogramming to combat against hypoxia-induced damage. It is well documented that activation of the HIF-1α protects the heart from ischemia or I/R induced injury [8] [7, 15]. Notably, partial depletion of HIF-1α completely abolishes the ischemic preconditioning-induced cardioprotection in HIF-1α^+/−^ mice [34], suggesting that the abundance of HIF-1α protein is a determining factor for its cardioprotective effect. It has been demonstrated that PHD family proteins play a central role in the regulation of the HIF-1α stability[35]. When oxygen availability is limited, PHD enzymatic activity will be inhibited, resulting in stabilization of HIF-1α protein and activation of HIF-1 pathway [35]. Although PHD2 is the major HIF-1α prolyl-4 hydroxylase in the heart and depletion of PHD3 alone has no effect on cardiac HIF-1α protein level at physiological condition, PHD3 has been suggested to play a compensative role in the regulation of HIF-1α stability, especially when PHD2 is absent or under ischemic condition [16, 17, 36]. Therefore, it is not surprising that we observed an increased induction of HIF-1α protein at hypoxia condition in PHD3 depleted cardiomyocytes. Since our data suggest that overexpression of HIF-1α protects cardiomyocytes from apoptosis induced by hypoxia, the increased stabilization of HIF-1α may partially contribute to the cardioprotection observed in PHD3 knockout mice.

Although activation of HIF pathway may be beneficial in treating or attenuating cardiac injury induced by ischemia or I/R, a growing body of evidence suggests that chronic activation of the HIF pathway also leads to cardiomyopathy [15, 37, 38]. In addition, activation of the HIF pathway has been implicated in many aspects of tumorigenesis because the same HIF-mediated pathways that are instrumental in cardioprotection (for example angiogenesis, anaerobic metabolism and cell survival) also promote survival of tumor cells [39]. For these reasons, caution must be taken when applying small-molecular inhibitors of PHDs to the treatment of ischemic heart diseases. In the meantime, there are a wide variety of signaling pathways that are independent of HIF pathway can be regulated by PHDs [40, 41]. We previously reported that PHD3 associated with and hydroxylated human homologue of *Caenorhabditis elegans* biological clock protein CLK-2 (HCLK2), an essential component of the ATR/Chk1/p53 pathway. The hydroxylation of HCLK2 is necessary for its interaction with ATR and the subsequent activation of ATR/Chk1 and the downstream p53. Inhibiting PHD3, either with the pan prolyl hydroxylase inhibitor DMOG or hypoxia, inhibits the activation of the ATR/Chk1 pathway but not ATM/Chk2 pathway and decreases apoptosis induced by DNA damage [20]. The data presented here suggest that this PHD3-dependent regulation on ATR/Chk1/p53 pathway is conserved in cardiomyocytes and it may be possible to develop PHD3 substrate-specific inhibitors that are capable of attenuating myocardial I/R injury without affecting HIF-mediated pathways.

Oxidative stress plays an important role in a wide range of heart diseases including myocardial infarction, hypertrophy and heart failure [42]. Pathologically high levels of ROS can damage cellular macromolecules such as lipids, proteins and DNA, which may eventually lead to the impairment of cardiac function [43]. Recently, it was reported that high levels of oxidative DNA damage and robust activation of the DDR are present in human hearts at end-stage cardiomyopathy, suggesting that DNA damage induced by chronic oxidative stress may contribute to the development of heart failure [44]. However, the role of the DDR induced by chronic oxidative stress observed in cardiomyopathy has not been well characterized. In contrast, acute and excessive production of ROS induced by doxorubicin was recently shown to activate the ATM/p53 pathway and promote cardiomyocyte apoptosis [32]. Partial depletion of p53 or treatment with antioxidant agents, which inhibit the activation of the ATM/p53 pathway, significantly attenuates cardiomyocyte apoptosis and contractile dysfunction [32]. p53 is a common downstream target of both the ATM/Chk2 and ATR/Chk1 pathways, the two major pathways of the DDR [45]. However, whether oxidative DNA damage activates the ATR/Chk1 pathway in cardiomyocytes is not known. Interestingly, our data demonstrate that both doxorubicin and H_2_O_2_ strongly activate Chk1, and inhibition of PHD3 dramatically inhibits the activation of Chk1, p53 and subsequent apoptosis in cardiomyocytes. Considering the essential role of PHD3 in the ATR/Chk1/p53 pathway [20], it is plausible that the ATR/Chk1 pathway may also be activated by oxidative DNA damage and therefore may play an important role of in cardiomyocyte apoptosis induced by oxidative stress. Furthermore, our data demonstrates that hypoxia-reoxygenation activates the DDR in cardiomyocytes but can be inhibited with depletion or inhibition of PHD3, suggesting that DDR inhibitors such as ATM or ATR specific inhibitors may also hold promise in ameliorating cardiac damage associated with hypoxia-reoxygenation injury.

## Supplementary Material

supplement

## Highlights

- Depletion of PHD3 attenuates myocardial injury induced by ischemia-reperfusion.
- Depletion of PHD3 further stabilizes HIF-1α at hypoxic condition.
- HIF-1α inhibits cardiomyocyte apoptosis induced by hypoxia.
- Depletion of PHD3 inhibits Chk1/p53 activation induced by oxidative stress.
- Depletion of PHD3 inhibits cardiomyocyte apoptosis induced by oxidative stress.

## Non-standard Abbreviations and Acronyms

PHD: prolyl-4 hydroxylase domain
LAD: left anterior descending
I/R: Ischemia-reperfusion
HIF: hypoxia inducible factor
DDR: DNA damage response
MI: myocardial infarction
AAR: area at risk
TTC: triphenyl tetrazolium chloride
ATM: ataxia telangiectasia mutated
ATR: ATM and Rad3-related
PKM2: pyruvate kinase M2
HCLK2: human homologue of the caenorhabditis elegans biological clock protein CLK-2
ROS: reactive oxygen species
Brdu: 5-bromo-2′-deoxyuridine
DMOG: dimethyloxaloylglycine
