# Supplementary material for: Depletion of PHD3 Protects Heart from Ischemia/Reperfusion Injury by Inhibiting Cardiomyocyte Apoptosis
Source: J Mol Cell Cardiol. Author manuscript; Available in PMC 2016 Mar 1. (PMC4374643; doi:10.1016/j.yjmcc.2015.01.007)
Supplement: supplement [file NIHMS660838-supplement.doc]

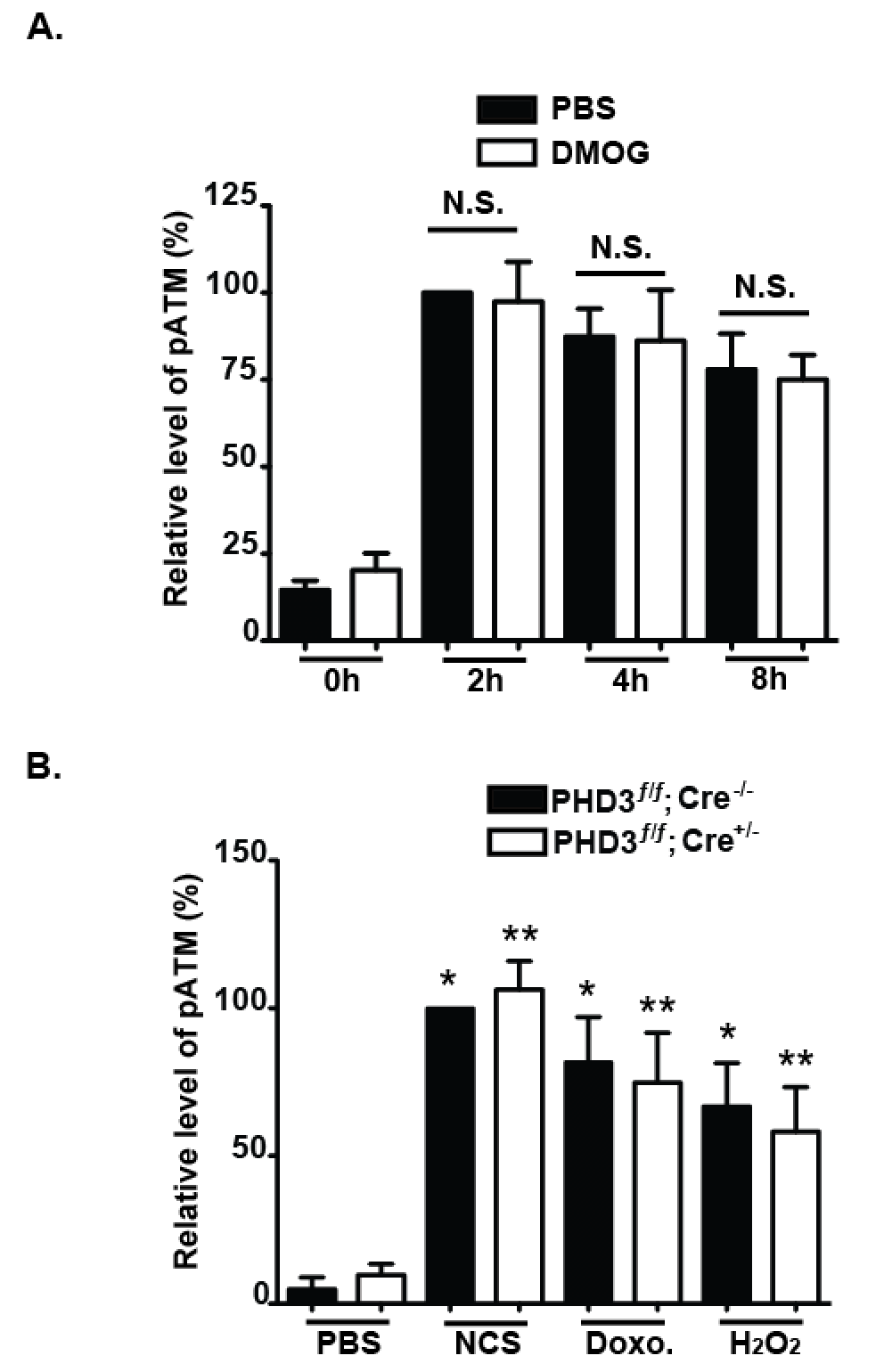


**Figure S1. DMOG or depletion of PHD3 has no effect on ATM activation. (A)** Neonatal rat ventricular myocytes were pre-treated with DMOG for 4h and then treated with doxorubicin (1μM) as indicated. Western blots were performed with anti-ATM or anti-phospho-ATM antibodies. Densitometry analyses were performed from three independent experiments. **(B)** Neonatal mouse ventricular myocytes (NMVMs) from PHD3^ƒ/ƒ^; Cre^+/-^ or PHD3^ƒ/ƒ^; Cre^-/-^ mice were treated with 4-hydroxyl-tamoxifen for 3 days. Cells were then treated with NCS, Doxorubicin or H_2_O_2_ for 1h and western blots were performed with anti-ATM or anti-phospho-ATM antibodies. Densitometry analyses were performed from three independent experiments. *p < 0.01, compared to NMVM from PHD3^ƒ/ƒ^; Cre^-/-^ mice treated with PBS; **p < 0.01, compared to NMVM from PHD3^ƒ/ƒ^; Cre^+/-^ treated with PBS.


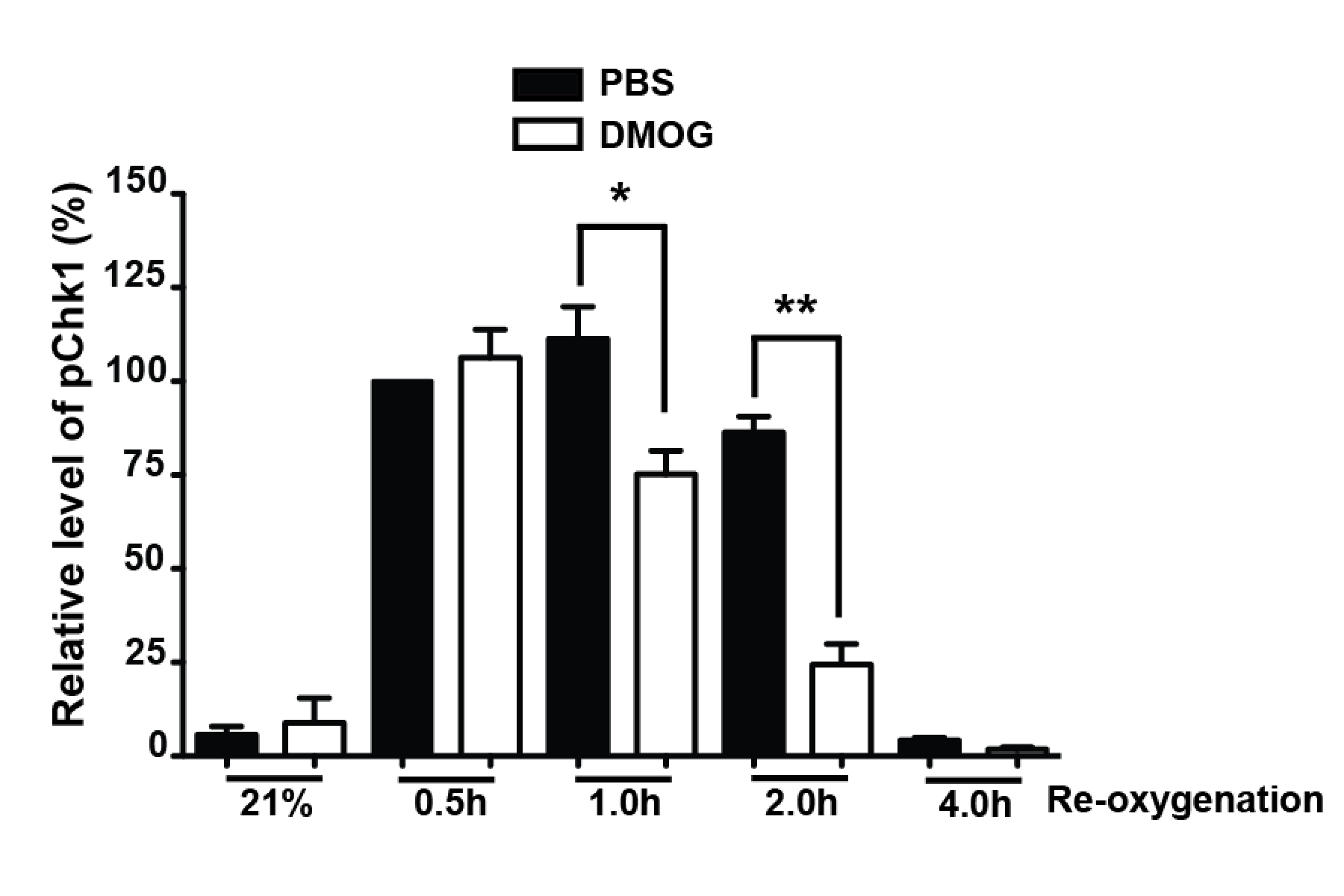


**Figure S2. Quantitative analysis of pChk1 upon re-oxygenation in the presence or absence of DMOG.** HL-1 cells were cultured in a hypoxia chamber for 6 hours and then switched to normoxic conditions for the indicated time with or without pretreatment with DMOG. Western blots were performed with anti-Chk1 or anti-phospho-Chk1 antibodies. Densitometry analyses were performed from three independent experiments. *p < 0.05; **p < 0.01.
